# Rhodomyrtone Accumulates in Bacterial Cell Wall and Cell Membrane and Inhibits the Synthesis of Multiple Cellular Macromolecules in Epidemic Methicillin-Resistant *Staphylococcus* *aureus*

**DOI:** 10.3390/antibiotics10050543

**Published:** 2021-05-07

**Authors:** Ozioma F. Nwabor, Sukanlaya Leejae, Supayang P. Voravuthikunchai

**Affiliations:** 1 Division of Infectious Diseases, Department of Internal Medicine, Faculty of Medicine, Prince of Songkla University, Hat Yai, Songkhla 90112, Thailand; nwaborozed@gmail.com; 2 Division of Biological Science, Faculty of Science and Natural Product Research Center of Excellence, Prince of Songkla University, Hat Yai, Songkhla 90112, Thailand; rhodomyrtone_sl@yahoo.com

**Keywords:** rhodomyrtone, antimicrobial resistance, macromolecule biosynthesis, methicillin-resistant *Staphylococcus* *aureus*

## Abstract

As the burden of antibacterial resistance worsens and treatment options become narrower, rhodomyrtone—a novel natural antibiotic agent with a new antibacterial mechanism—could replace existing antibiotics for the treatment of infections caused by multi-drug resistant Gram-positive bacteria. In this study, rhodomyrtone was detected within the cell by means of an easy an inexpensive method. The antibacterial effects of rhodomyrtone were investigated on epidemic methicillin-resistant *Staphylococcus* *aureus*. Thin-layer chromatography demonstrated the entrapment and accumulation of rhodomyrtone within the bacterial cell wall and cell membrane. The incorporation of radiolabelled precursors revealed that rhodomyrtone inhibited the synthesis of macromolecules including DNA, RNA, proteins, the cell wall, and lipids. Following the treatment with rhodomyrtone at MIC (0.5–1 µg/mL), the synthesis of all macromolecules was significantly inhibited (*p* ≤ 0.05) after 4 h. Inhibition of macromolecule synthesis was demonstrated after 30 min at a higher concentration of rhodomyrtone (4× MIC), comparable to standard inhibitor compounds. In contrast, rhodomyrtone did not affect lipase activity in staphylococci—both epidemic methicillin-resistant *S.* *aureus* and *S*. *aureus* ATCC 29213. Interfering with the synthesis of multiple macromolecules is thought to be one of the antibacterial mechanisms of rhodomyrtone.

## 1. Introduction

Methicillin-resistant *Staphylococcus* *aureus* is a significant cause of morbidity and mortality worldwide in both hospital- and community-acquired infections [1]. It causes an array of diseases, ranging from minor localized skin lesions to life-threatening deep tissue damage and systemic infections such as pneumonia, endocarditis, osteomyelitis, arthritis, sepsis, and toxic shock syndrome. The spread of isolates resistant to available antibiotics has limited therapeutic options for the treatment and control of infections caused by MRSA [2]. Due to the severity and spread of MRSA, and the intense health and economic burdens associated with its infections, MRSA have been classified in the second tier of the priority pathogen list [3]. The current management of MRSA-mediated bacteremia depends on the administration of vancomycin, daptomycin, or newer agents, including teicoplanin, telavancin, ceftaroline, oxazolidinones, and tigecycline [4,5]. The use of antibiotic combination therapies has been highlighted as a treatment option against MRSA [6,7] and other multidrug-resistant bacteria [8,9]. However, the effective management of MRSA requires collaborative actions, and the development of a novel antibacterial agent with a new mode of action. Unfortunately, due to the lag in the discovery of novel bioactive compounds, the eradication of infectious diseases remains elusive.

As the scourge of antimicrobial resistance continues to plague humanity, with daunting consequences, the search for alternative treatment options to effectively manage and control infections caused by drug-resistant bacteria has become inevitable. Several strategies have been adopted, including the use of natural products like plant phytochemicals [10,11,12] and microbial metabolites [13,14]. Studies have demonstrated the excellent antibacterial activities of *R. tomentosa* against Gram-positive bacterial isolates, including drug-resistant pathogenic isolates. Transmission electron microscopy revealed that *R. tomentosa* extract altered the morphology of MRSA, leading to cell lysis [15]. The excellent antibacterial potential of this plant have been linked to its primary active agents, namely, “rhodomyrtone [16] and its derivative compounds”.

Rhodomyrtone, an acylphloroglucinols compound, has exhibited significant antibacterial activity, equivalent to vancomycin (MIC = 0.5/1 µg/mL), against a wide range of Gram-positive bacteria, including *Streptococcus mutans* [17], *S. pyogenes* [18], *Clostridium difficile* [19], etc. At subinhibitory concentrations, rhodomyrtone treatment induced prominent changes, with alterations to the cell wall of MRSA, resulting in cell disintegration and lysis. Furthermore, proteomic studies revealed that rhodomyrtone affected various metabolic pathways in MRSA, resulting in changes in cellular and extracellular proteins associated with cell division, protein degradation, oxidative stress response, and virulence factors [20]. Membrane proteins were trapped in vesicles, with increased fluidity following the treatment of cells with rhodomyrtone [21]. In addition, the expression of gene-encoding proteins involved in amino acid metabolism, membrane function, ATP-binding cassette (ABC) transportation, and lipoprotein and nucleotide metabolism in MRSA was affected by rhodomyrtone treatment [22].

Thus, in this study we aimed to further investigate the antibacterial effects of rhodomyrtone on the biosynthesis of cellular macromolecules of *Staphylococcus aureus*. The accumulation of rhodomyrtone was investigated using thin-layer chromatography, and the effects of rhodomyrtone on the synthesis of cellular macromolecules were investigated using radiolabelled standard inhibitors.

## 2. Results and Discussion

### 2.1. Antibacterial Effects of Rhodomyrtone and Other Antimicrobial Agents

The minimum inhibitory and minimum bactericidal concentrations of the antimicrobial agents used as standard macromolecule inhibitors are elucidated in Table 1. The MICs and MBCs of the standard macromolecule inhibitors on epidemic methicillin-resistant *S. aureus* (EMRSA-16) and *S*. *aureus* ATCC 29213 ranged from 0.015–16 and 0.06–128 µg/mL, respectively. The minimum inhibitory and minimum bactericidal concentrations of rhodomyrtone were previously reported to be 0.5/0.5 µg/mL for EMRSA-16 and 0.5/1 µg/mL for *S. aureus* ATCC 29213 [23]. The antibacterial activities of rhodomyrtone have previously been demonstrated against several Gram-positive bacterial isolates, including *S. aureus* and *S. epidermidis* (MIC = 0.25–2 µg/mL) [24], *Propionibacterium acnes* (MIC = 0.12–0.5 µg/mL) [25], *Streptococcus pneumoniae* (MIC = 0.125–1 µg/mL) [26], *S. aureus* (MIC = 1.83 µg/mL) [27], *Streptococcus* *suis* (MIC = 0.5 µg/mL) [28], and *Clostridium difficile* (MIC = 0.625–2.5 µg/mL) [19]. The antibacterial activity of rhodomyrtone against *P. acnes* was reported to be comparable with that of the standard antibiotic erythromycin [25], and with that of vancomycin for *S. aureus,* and *C. difficile* [19,23].

### 2.2. Localisation of Rhodomyrtone in Pathogenic Bacteria

**Figure 1:**
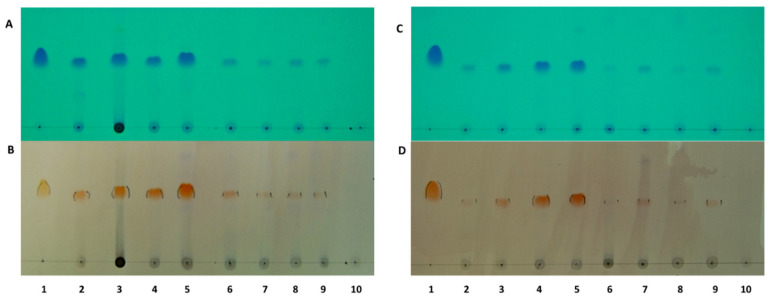
Localization of rhodomyrtone in methicillin-resistant *Staphylococcus aureus* (EMRSA-16) (**A**,**B**) and ATCC 29213 (**C**,**D**) as determined using the thin-layer chromatography technique. The bacterial cells were treated with rhodomyrtone at 4× MIC for 1–4 h. Rhodomyrtone was visualized under UV absorption at 254 nm (**A**,**C**) and after spraying with anisaldehyde/H_2_SO_4_ and heating (**B**,**D**). Lanes 2–5 are fractions from the cell wall and cell membrane of rhodomyrtone-treated cells, whereas lanes 6–9 are fractions of cytoplasm from the treated cells. One-percent DMSO was used as a negative control. Lane 1: reference rhodomyrtone; lanes 2 and 6: treatment for 1 h; lanes 3 and 7: treatment for 2 h; lanes 4 and 8: treatment for 3 h; lanes 5 and 9: treatment for 4 h; lane 10: negative control.

To investigate the antibacterial mechanisms and targets of rhodomyrtone in EMRSA-16 and *S.* *aureus* ATCC 29213, primary screening of rhodomyrtone accumulation in the bacterial cells was performed. The results indicated that rhodomyrtone was present in the cell wall and cell membrane of EMRSA-16 and ATCC 29213 cells after treatment with 4× MIC for 1–4 h (Figure 1). The compound displayed a dark blue colouration on TLC plates under UV absorption at 254 nm and an orange colour after spraying with anisaldehyde/H_2_SO_4_ reagent and heating. The results demonstrated that rhodomyrtone was trapped in the cell wall and cell membrane of the test bacterial strains. In addition, the time of treatment affected the accumulation, as demonstrated by the higher levels of accumulated compound over time. For both bacterial strains (EMRSA and *S.* *aureus* ATCC 29213), 4 h treatment showed a higher accumulation of rhodomyrtone in the cell wall and cell membrane. Lower levels of rhodomyrtone were observed in the cytoplasmic fraction of the rhodomyrtone-treated cells. The accumulation of rhodomyrtone in the cell wall and cell membrane might be due to the interaction of rhodomyrtone with membrane proteins. In addition, the high molecular mass of rhodomyrtone (442.5 g/mol) might affect the diffusion of rhodomyrtone, leaving it entrapped within the cell wall and cell membrane. Previous studies have demonstrated that rhodomyrtone is a membrane-active compound [29]. The interaction of rhodomyrtone with the bacterial cell wall and membrane resulted in membrane invagination, which has been demonstrated as possible mechanism of rhodomyrtone mediated membrane distortion [21,28]. The presence of rhodomyrtone in cytoplasmic extracts might be due to the capability of rhodomyrtone to penetrate the cytoplasm of the cells. In addition, scanning electron micrography carried out in previous studies indicated membrane fragmentation with physical and morphological changes [30].

### 2.3. Effects of Rhodomyrtone on Macromolecular Biosynthesis

**Figure 2:**
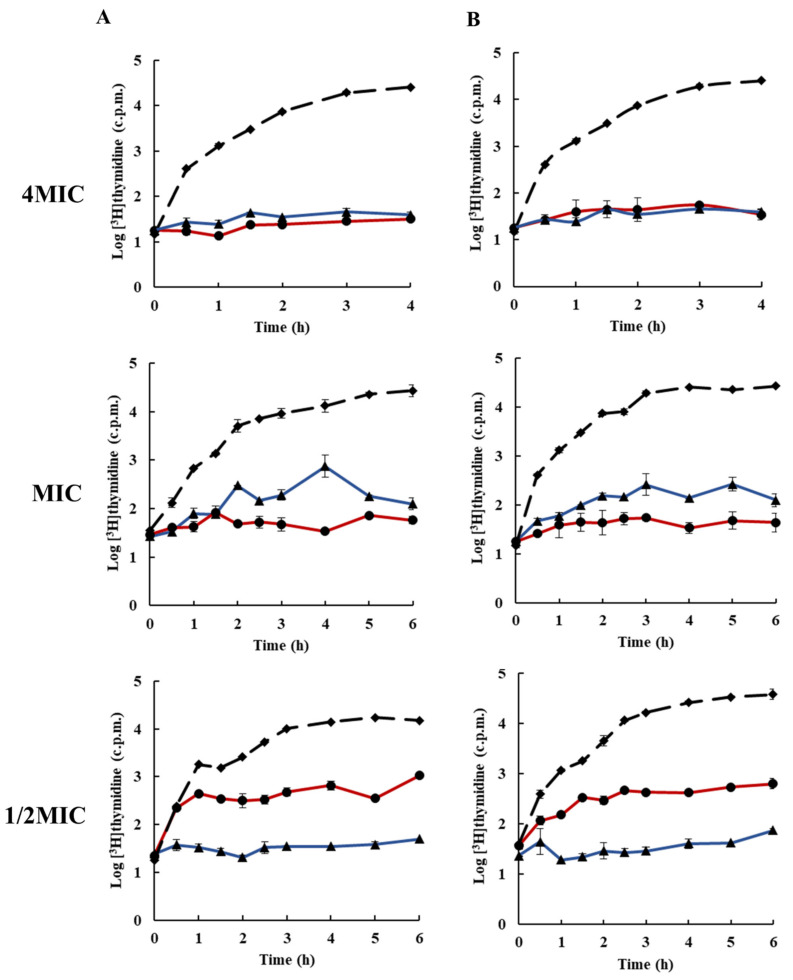
Effects of rhodomyrtone on DNA synthesis at 4× MIC, MIC, and 1/2× MIC on (**A**) EMRSA-16, (**B**) *S. aureus* ATCC 29213. DNA synthesis was determined by incorporation of [^3^H]thymidine, rhodomyrtone (blue ▲), ELB21 (red ●), and 1% DMSO (broken line ◆). Each symbol indicates the mean ± SD for duplicates.

**Figure 3:**
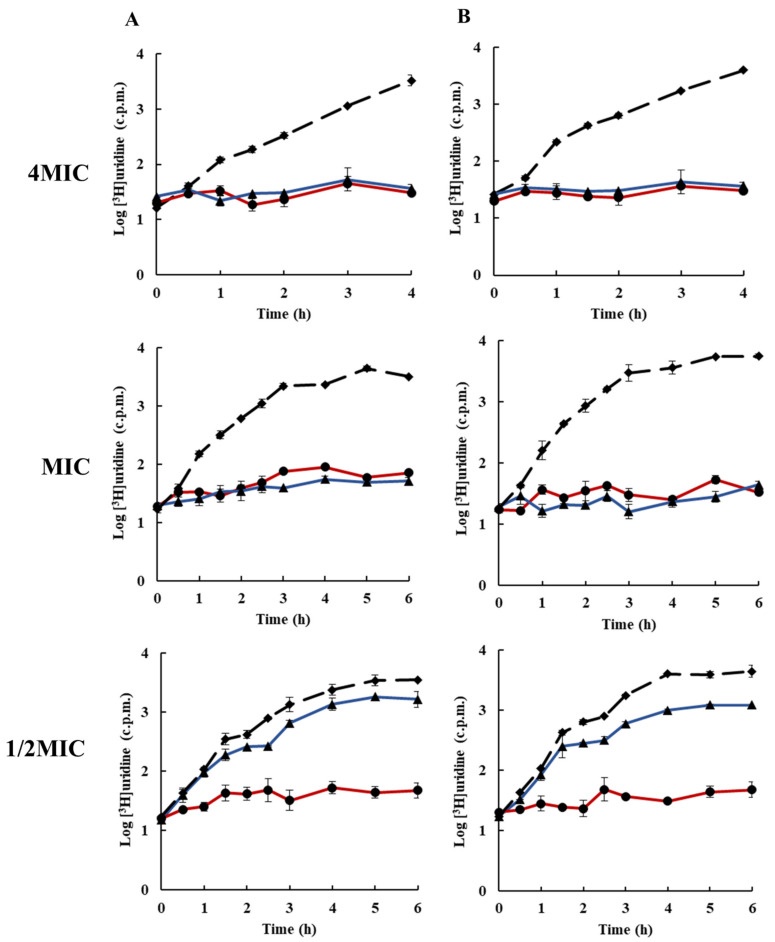
Effects of rhodomyrtone on RNA synthesis at 4× MIC, MIC, and 1/2× MIC on (**A**) EMRSA-16, (**B**) *S. aureus* ATCC 29213. RNA synthesis, determined by incorporation of [^3^H]uridine, rhodomyrtone (blue ▲),rifampicin (red ●), and 1% DMSO (broken line ◆). Each symbol indicates the mean ± SD for duplicates.

To elucidate the molecular mechanism of action of rhodomyrtone, the inhibitory activity of the compound on bacterial macromolecular synthesis was monitored by means of the incorporation of radiolabelled molecules. Macromolecular synthesis assays can be used to determine the global mode of action of an inhibitor on the growth of bacteria. The effects of rhodomyrtone on macromolecule synthesis were compared with those of known and specific macromolecule inhibitors (DNA:ELB21), (RNA:rifampicin), (protein:chloramphenicol), (cell wall:vancomycin), and (lipid:platensimycin), each at 1/2× MIC (0.25 µg/mL), MIC (0.5 µg/mL), and 4× MIC (2 µg/mL). Figure 2 and Figure 3 illustrates the effects of rhodomyrtone at 4× MIC, MIC, and 1/2MIC on nucleic acid biosynthesis. The results indicated that DNA biosynthesis was inhibited at bactericidal and bacteriostatic concentrations (4×MIC and MIC). At 1/2× MIC, DNA biosynthesis was reduced by <1log after 3–6 h exposure to rhodomyrtone. Similar results were obtained for both tested bacterial strains. In addition, the effects of rhodomyrtone were similar to that of the standard DNA inhibitor compound. However, at MIC, rhodomyrtone displayed better activity when compared with the standard DNA inhibitor compound. For both test bacterial strains, 4× MIC and MIC rhodomyrtone inhibited RNA synthesis at all the tested times. However, at 1/2 MIC, RNA synthesis was not inhibited but slightly reduced. A previous study reported that rhodomyrtone treatment inhibited the transcription of staphylococcal biofilm-related genes [31]. Transcriptomic analysis of rhodomyrtone-treated cells further revealed that the compound induced changes in the gene transcription of MRSA, resulting in the alteration of expression of several functional classes of bacterial proteins [22].

**Figure 4:**
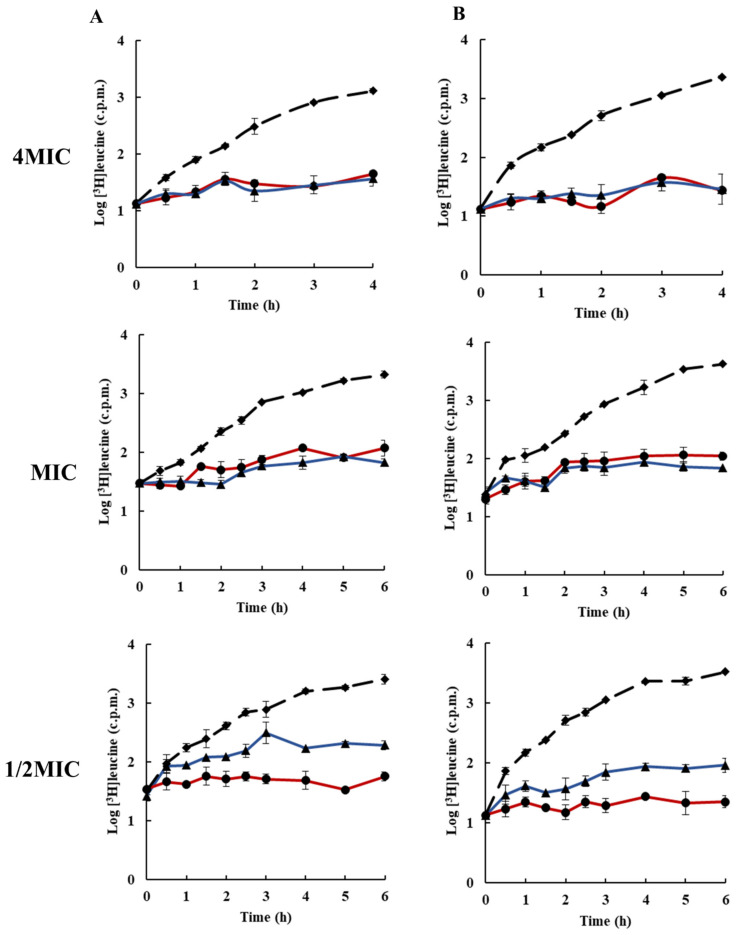
Effects of rhodomyrtone on protein synthesis at 4× MIC, MIC, and 1/2× MIC on (**A**) EMRSA-16 and (**B**) *S. aureus* ATCC 29213. Protein synthesis, determined by incorporation of [^3^H]leucine, rhodomyrtone (blue ▲),chloramphenicol (red ●), and 1% DMSO (broken line ◆). Each symbol indicates the mean ± SD for duplicates.

**Figure 5:**
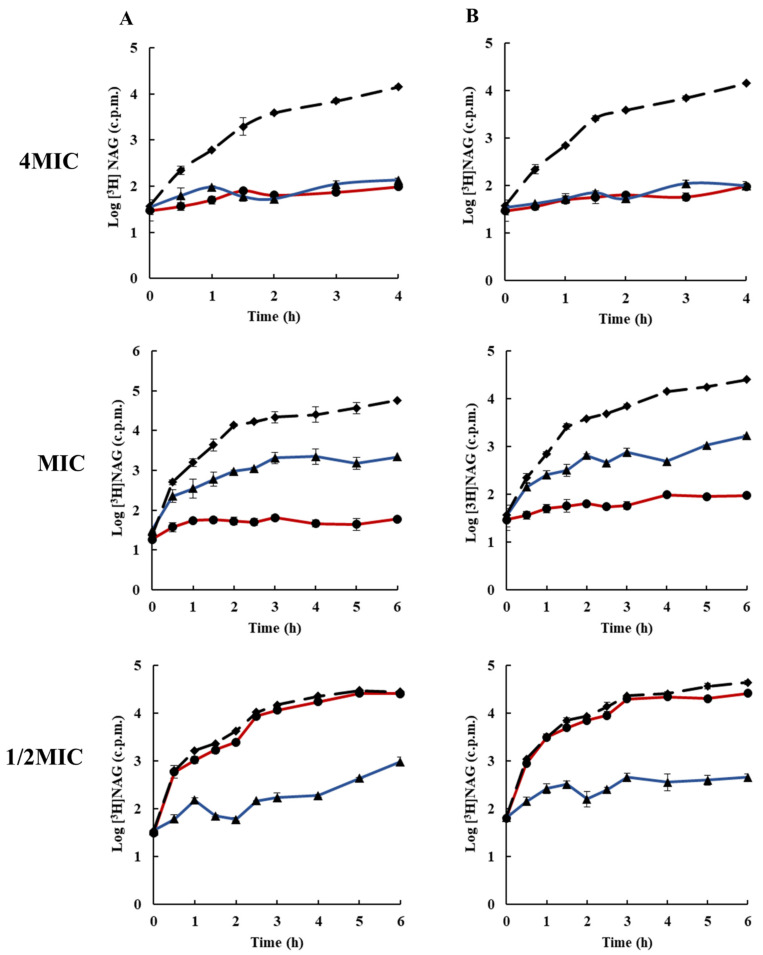
Effects of rhodomyrtone on cell wall synthesis at 4× MIC, MIC, and 1/2× MIC on (**A**) EMRSA-16 and (**B**) *S. aureus* ATCC 29213. Cell wall synthesis was determined by incorporation of N-acetyl-d-[1-^3^H]glucosamine. rhodomyrtone (blue ▲), vancomycin (red ●), and 1% DMSO (broken line ◆). Each symbol indicates the mean ± SD for duplicates.

**Figure 6:**
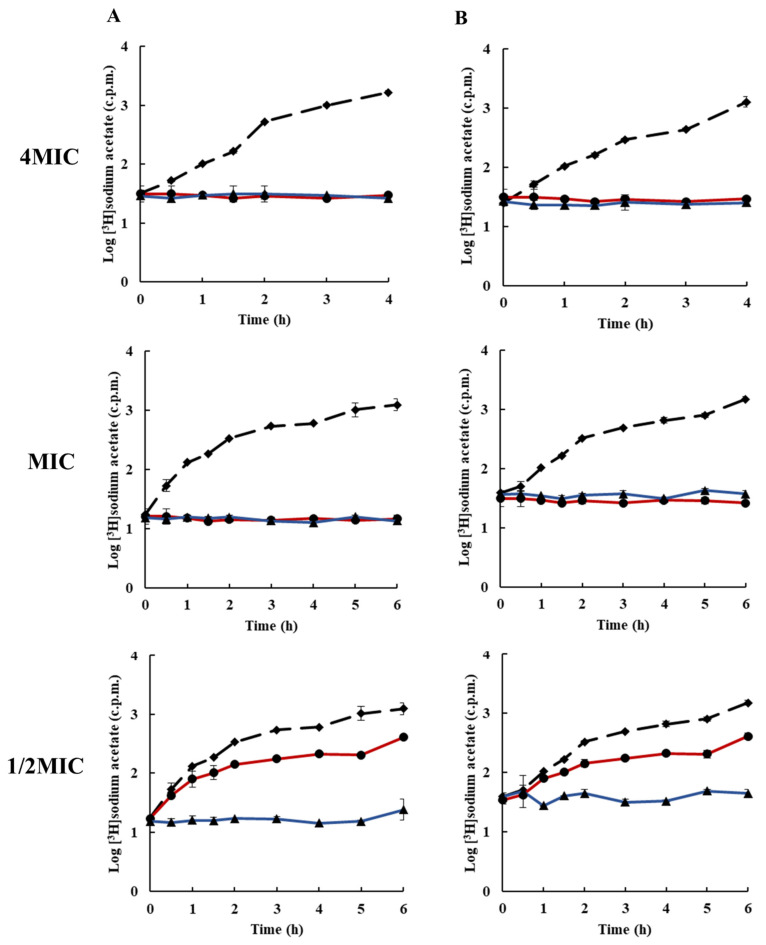
Effects of rhodomyrtone on lipid synthesis at 4× MIC, MIC, and 1/2× MIC on (**A**) EMRSA-16 and (**B**) *S. aureus* ATCC 29213. Lipid synthesis was determined by incorporation of [^3^H]sodium acetate, rhodomyrtone (blue ▲), platensimycin (red ●), and 1% DMSO (broken line ◆). Each symbol indicates the mean ± SD for duplicates.

The effects of rhodomyrtone on protein and cell wall synthesis is presented in Figure 4 and Figure 5. The results revealed that at 4× MIC, both protein and cell wall synthesis were completely inhibited. At MIC, the inhibition of protein synthesis by rhodomyrtone was similar to that of the standard inhibitor compound; however, rhodomyrtone at MIC showed better cell wall biosynthesis inhibition compared with the standard inhibitor compound. At 1/2× MIC, rhodomyrtone showed ≤ 1log inhibition of protein and cell wall synthesis. Previous studies revealed that rhodomyrtone is a membrane-active compound. Electron micrographs of rhodomyrtone-treated staphylococcal cells revealed membrane disruption with detachment of the cell wall from the cytoplasmic membrane and shrinking of cells [32]. In addition, using laurdan fluorescence spectroscopy it was observed that rhodomyrtone treatment induced a dramatic increase in the membrane fluidity of *B. subtilis* strains [21]. Figure 6 shows the effects of rhodomyrtone on lipid biosynthesis. The results indicated that rhodomyrtone at 4× MIC and MIC completely inhibited the lipid biosynthesis of *S. aureus* (EMRSA-16). The synthesis of all macromolecules was inhibited by ≥1log after treatment for 4 h. The compound at 4× MIC completely inhibited all macromolecules after 30 min. The antimicrobial mechanisms of rhodomyrtone have been investigated by several researchers and have been demonstrated to interfere with protein and nucleic acid synthesis [20,22,31]. Transcriptional analysis of MRSA exposed to subinhibitory concentrations of rhodomyrtone revealed interference with gene expression, resulting in the induction of 64 genes and repression of 35 genes, with changes in genes encoding essential proteins involved in amino acid metabolism, membrane function, ATP-binding cassette transportation, lipoprotein, and nucleotide metabolism [22]. An incomplete nucleoid segregation and septum misplacement was observed in *Streptococcus suis* after treatment with rhodomyrtone [28]. Rhodomyrtone has also been reported to interfere with the expression of virulence genes and to disrupt *sigB* [33]. Metabolic pathways involving amino acid synthesis, carbohydrate metabolism, lipid metabolism, and nucleotide metabolism were reportedly disrupted after rhodomyrtone treatment [20]. Furthermore, proteomic and metabolomic studies have revealed that rhodomyrtone interferes with several metabolic pathways, including amino acid biosynthesis, nucleic acid biosynthesis, carbohydrates, and lipid metabolism in *S. pneumoniae* [34].

### 2.4. Effect of Rhodomyrtone on the Extracellular Lipase Activity in Staphylococcus aureus

The ability of rhodomyrtone to inhibit lipase production by *S.* *aureus* was studied (Table 2). The results indicated that rhodomyrtone did not suppress the lipase activity of *S.* *aureus* compared with untreated *S.* *aureus*. At the tested concentrations, no significant difference in lipase production was observed between treated and untreated cells. This indicates that although the compound could obstruct the process of lipid synthesis, it did not affect the induction of lipase activity. Lipase production is an important virulence mechanism in the pathogenesis of *S. aureus.* Thus, the inhibition of staphylococcal lipase activity might be employed as an anti-virulent strategy for the management of infections caused by pathogenic *S. aureus.* Although the results of this study indicate that the rhodomyrtone did not inhibit the lipase production of the tested strains, Wunnoo et al. [35], reported that rhodomyrtone treatment significantly decreased both lipase and protease enzymes in *Propionibacterium acnes*.

## 3. Materials and Methods

### 3.1. Antibiotics and Chemicals

Reagents used were purchased as follows: Mueller-Hinton agar (MHA) and Mueller-Hinton broth (MHB) from Oxide (Hampshire, UK); dimethyl sulfoxide (DMSO), chloramphenicol, rifampicin, vancomycin, and trichloroacetic acid (TCA) from Sigma-Aldrich (St. Louis, MO, USA); ethanol, methanol, chlorophorm, sodium chloride, and scintillation counter liquid from Fisher Scientific (Leicestershire, UK); and platensimycin from Calbiochem (Darmstade, Germany). The following radiolabelled chemicals were obtained from Perkin Elmer (California, CA, USA): [6-^3^H]thymidine, >97%, 250 µCi (9.25 MBq); [5-^3^H]uridine, >97%, 250 µCi (9.25 MBq); L-[4,5-^3^H(N)]leucine, >97%, 250 µCi (9.25 MBq); N-acetyl-d-[1-^3^H]glucosamine (NAG), 250 µCi (9.25 MBq); acetic acid, sodium salt, [^3^H]-specific activity: 2–5 Ci (74.0–185 GBq)/mMole, 5 mCi (185 MBq). All standard chemicals were of analytical grade. Purified rhodomyrtone was isolated as previously described [36,37]; the purity of the compound was confirmed by comparing it to nuclear magnetic resonance and mass spectrometry references [38,39].

### 3.2. Bacterial Strains and Growth Conditions

Epidemic MRSA-16 and *S. aureus* ATCC 29213 were obtained from the collection of the Laboratory of Microbiology, School of Pharmacy, University College London, UK. All bacterial strains were grown in MHB and incubated at 37 °C for 3–5 h with aeration, and turbidity was adjusted to McFarland standard number 0.5 (~1.5 × 10^8^ colony-forming unit (CFU/mL).

### 3.3. Antibacterial Assay

A modified broth microdilution method [40] was adopted to investigate antibacterial activities. Briefly, rhodomyrtone and antibiotics (chloramphenicol, pyrrolobenzodiazepine dimer ELB-21, platensimycin, rifampicin, and vancomycin) were diluted twofold to final concentrations ranging from 0.03125–256 µg/mL. One hundred microliters of the bacterial culture (~10^6^ CFU/mL) were inoculated in 80 µL of MHB, supplemented with 20 µL of rhodomyrtone or antibiotics. The microtiter plates were incubated at 37 °C for 16–18 h. Cultures treated with 1% DMSO were used as controls. The minimum inhibitory concentration (MIC) and minimum bactericidal concentration (MBC) were recorded. The experiment was performed in triplicate.

### 3.4. Primary Screening for Rhodomyrtone Target in Staphylococcus aureus

Localisation of rhodomyrtone in EMRSA-16 and *S. aureus* ATCC 29213 was performed as previously reported [23]. In brief, actively growing bacterial culture was adjusted to McFarland standard number 4 (~1.2 × 10^9^ CFU/mL). Then, rhodomyrtone (4× MIC) was added to the cultures and incubated at 37 °C. Afterwards, 10 mL of cultures were collected at 0, 1, 2, 3, and 4 h, centrifuged and lysed by sonification. The pellets (cell wall and cell membrane) and supernatant (cytoplasm) were extracted with ethyl acetate. Localisation of rhodomyrtone in the pathogens was monitored by means of thin-layer chromatography (TLC). The experiment was performed in triplicate.

### 3.5. Inhibition of Macromolecular Biosynthesis

Macromolecular biosynthesis assays were performed on cultures of EMRSA-16 and *S.* *aureus* ATCC 29123 as previously described by [41] with some modifications. DNA, RNA, protein, cell wall, and lipid synthesis in the mid-exponential phase of bacteria cultures (~10^8^ CFU/mL in MHB) were monitored through the incorporation of the radiolabelled precursors [^3^H] thymidine, [^3^H]uridine, [^3^H]leucine, [^3^H]NAG, and [^3^H]sodium acetate, respectively. The cultures were added to universal test tubes containing rhodomyrtone at 4× MIC, MIC, and 1/2× MIC. The radiolabelled precursors were added at a final concentration of 1 µCi/mL. Cultures were incubated at 37 °C with constant shaking (200 rpm). Samples (350 µL) were withdrawn at intervals and the reaction was terminated by adding 350 μL ice-cold 10% TCA. The mixtures were then centrifuged at 20,000 rpm at 4 °C for 15 min. Supernatants were discarded and pellets were washed twice with 500 µL ice-cold 5% TCA. The pellets were dissolved in 200 µL distilled water, transferred to scintillation vials, and then added up to 4 mL scintillation fluid. The samples were read with a scintillation counter. ELB21 (DNA), rifampicin (RNA), chloramphenicol (protein), and vancomycin (cell wall) were used as positive control agents for macromolecular synthesis inhibition assays. For lipid synthesis, the bacterial cells in the mid-exponential growth phase were added to falcon tubes containing rhodomyrtone at 4× MIC, MIC, and 1/2× MIC. [^3^H] sodium acetate at 0.2 µCi/mL was added to the mixtures, and the reactions were incubated with shaking at 37 °C. Samples (1000 µL) were withdrawn at intervals, and 3750 µL of chloroform/methanol (1:2) was added to stop the reaction. The mixture was vortexed for 10 min, chloroform (1250 µL) was added to the mixture, vortexed for 2 min, followed by addition of 1250 µL distilled water and vortexed for 1 min. The mixtures were centrifuged at 13,000 rpm for 10 min, and 500 µL of organic phase was transferred to a scintillation vial and allowed to evaporate to dryness in a fume hood. The samples were then counted using a scintillation counter. A negative control was used with 1% DMSO. All experiments were carried out in triplicate.

### 3.6. Lipase Activity Assay

The effect of rhodomyrtone on the lipase activity of *S*. *aureus* ATCC 29213 was investigated as previously reported with some modifications [42]. Lipase activity in the presence of rhodomyrtone at 1/2× MIC and 1/4× MIC were estimated on MHA, supplemented with 1% tributyrin. Mid-exponential phase culture was centrifuged at 5000 rpm for 10 min and the pellet was adjusted to a 0.5 McFarland standard. An aliquot of 5 µL of the culture was spot-inoculated in radial patterns on the agar plate surfaces and incubated at 37 °C for 3 days. The inhibition of enzymatic activity was evaluated as total when the halo was absent and partial when the halo was ≤ 50%, compared with the positive control. The experiment was performed in triplicate.

### 3.7. Statistical Analysis

Each experiment was performed in triplicate, and experimental values were expressed as means ± SD. The statistical significance of the differences between the control (DMSO-treated) and test (rhodomyrtone- and standard inhibitor-treated) cells were analyzed using one-way analysis of variance (ANOVA). The Statistical Package for the Social Sciences 20 (IBM Incorporated, Armonk, NY, USA) was used for the analysis at a 95% confidence interval (Scheffe test at *p* < 0.05).

## 4. Conclusions

Rhodomyrtone, a potent plant-derived antibacterial compound, has demonstrated membrane-active effects against several pathogenic Gram-positive bacterial isolates. Although several studies have demonstrated the effectiveness of rhodomyrtone in the inactivation of microbial cells, its mode of action is still not conclusively understood. In this study, we investigated the mechanism of action of rhodomyrtone by evaluating for rhodomyrtone accumulation in bacterial cell walls and the cell membrane and assessing its effects on the synthesis of cellular macromolecules. This study demonstrated that rhodomyrtone accumulates in the bacterial cell wall and the cell membrane and inhibits the synthesis of multiple cellular macromolecules, including nucleic acid, proteins, the cell wall, and lipids. The results revealed that rhodomyrtone targets multiple cellular components in MRSA and thus might be an alternative antibacterial agent, effective for the management of methicillin and other drug-resistant Gram-positive bacteria.

## Figures and Tables

**Table 1:** Minimum inhibitory concentrations and minimum bactericidal concentrations of rhodomyrtone and standard macromolecule inhibitors on *Staphylococcus aureus* ATCC 29213 and epidemic methicillin-resistant *Staphylococcus aureus* (EMRSA-16).

Antimicrobial Agent	MIC/MBC (µg/mL)	
	*S*. *aureus* ATCC 29213	EMRSA-16
Rhodomyrtone	0.5/1	0.5/0.5
Chloramphenicol	16/32	8/128
ELB21	0.0625/0.125	0.0625/0.125
Platensimycin	1/>8	1/>8
Rifampicin	0.015/0.06	0.0625/0.125
Vancomycin	1/1	1/1

**Table 2:** Effects of rhodomyrtone on staphylococcal lipase activity, measured as zone diameter on MHA supplemented with 1% tributyrin.

Time (h)	Zone Diameter (mm)			
	Untreated	0.5× MIC	1× MIC	4× MIC
0	9.0	8.5	7.0	7.7
1	17.0	16.7	17.3	19.0
2	18.6	16.3	15.5	16.7
3	16.7	15.7	15.7	16.0
4	17.0	14.7	16.7	17.0

## Data Availability

All data were included in the manuscript.
